# Acute Physical Exercise Can Influence the Accuracy of Metacognitive Judgments

**DOI:** 10.1038/s41598-019-48861-3

**Published:** 2019-08-27

**Authors:** Matthew A. Palmer, Kayla Stefanidis, Ashlee Turner, Peter J. Tranent, Rachel Breen, Talira Kucina, Laura Brumby, Glenys A. Holt, James W. Fell, James D. Sauer

**Affiliations:** 1 Department of Psychology, School of Medicine, University of Tasmania, Hobart, Australia; 2 Thompson Institute, University of the Sunshine Coast, Birtinya, Australia; 3 School of Psychology, University of Sydney, Sydney, Australia; 4 Department of Psychology, University of Chester, Chester, UK; 5 School of Health Science, University of Tasmania, Hobart, Australia

**Keywords:** Psychology, Human behaviour

## Abstract

Acute exercise generally benefits memory but little research has examined how exercise affects metacognition (knowledge of memory performance). We show that a single bout of exercise can influence metacognition in paired-associate learning. Participants completed 30-min of moderate-intensity exercise before or after studying a series of word pairs (*cloud*-*ivory*), and completed cued-recall (*cloud*-?; Experiments 1 & 2) and recognition memory tests (*cloud*-? *spoon; ivory*; *drill*; *choir*; Experiment 2). Participants made judgments of learning prior to cued-recall tests (JOLs; predicted likelihood of recalling the second word of each pair when shown the first) and feeling-of-knowing judgments prior to recognition tests (FOK; predicted likelihood of recognizing the second word from four alternatives). Compared to no-exercise control conditions, exercise before encoding enhanced cued-recall in Experiment 1 but not Experiment 2 and did not affect recognition. Exercise after encoding did not influence memory. In conditions where exercise did not benefit memory, it increased JOLs and FOK judgments relative to accuracy (Experiments 1 & 2) and impaired the relative accuracy of JOLs (ability to distinguish remembered from non-remembered items; Experiment 2). Acute exercise seems to signal likely remembering; this has implications for understanding the effects of exercise on metacognition, and for incorporating exercise into study routines.

## Introduction

Ample evidence indicates that exercise enhances cognition. This is true not only of sustained, long-term exercise programs^1,2^ but also acute bouts of exercise—the focus of the present research. It is well-established that a single bout of exercise can enhance performance on many cognitive tasks, including attention, executive function, motor-task memory, and short-term and long-term memory^3–8^. Predominant theoretical accounts of these effects hold that exercise influences various neurobiological mechanisms that support cognition and memory. For example, acute exercise increases levels of brain-derived neurotrophic factor (BDNF), which supports neuronal development and plasticity^9,10^. It also increases the concentration of neurotransmitters such as dopamine, epinephrine, and norepinephrine, which support the consolidation and regulation of memory^4,11,12^.

We examined whether an acute bout of moderate intensity exercise affects metacognition, the ability to accurately monitor and evaluate one’s knowledge and memory^13,14^. One important aspect of metacognition is predicting the likelihood that studied material will be remembered in the future (i.e., making *judgments of learning*; JOLs)^15^. Good metacognitive monitoring allows people to gauge accurately what they will remember and what they will not. This is crucial to many tasks. For example, a student preparing for an exam must judge what content has been learned adequately and what content requires more study. A witness to a crime must judge whether he or she will be able to remember important details (e.g., a car license number or the appearance of the culprit) or whether more attention must be paid to these things. In many domains, metacognitive judgments are made before, after, or during acute physical exercise. Consider a student studying immediately after a gym session, or a police officer chasing a criminal on foot. In such situations, there are clear benefits for accurate metacognition. Understanding how exercise affects this ability will allow us to consider ways to compensate for any effects and, thus, maximize metacognitive accuracy. For example, if it emerges that exercise before study enhances memory without affecting metacognition, this would be worth knowing because it would allow students to exercise before studying without concern that exercise might warp their ability to gauge their own learning. If it emerges that exercise systematically decreases the accuracy of metacognitive judgments under certain circumstances, then by educating people about these effects, we can help people take them into account when evaluating memory decisions (e.g., by lowering expectations about likely memory accuracy).

Metacognition can be evaluated in terms of absolute and relative accuracy, which can vary independently of one another^16–18^. Absolute accuracy refers to the degree to which metacognitive judgments correspond to actual levels of memory performance. This can deviate from perfect in several ways, including overconfidence (whereby the predicted likelihood of remembering is, on average, higher than actual accuracy) and under-confidence (whereby predictions are lower than actual accuracy). Relative accuracy—sometimes termed *resolution* or *discrimination*—refers to the ability to distinguish items that will be remembered from items that will not. A person with perfect discrimination assigns high JOLs to all items that they successfully remember on a memory test and low JOLs to all items they fail to remember.

How might exercise affect metacognition? According to several theoretical accounts, factors that influence memory strength can influence the accuracy of metacognitive judgments. These accounts point to various different ways that effects of exercise on cognition might translate into effects on metacognition.

First, any beneficial effects of exercise on memory might translate to increased relative accuracy of metacognitive judgments. Theoretical accounts including the *optimality hypothesis*^19,20^ and *memory constraint hypothesis*^21^ hold that stronger memory allows one to not only make better memory decisions, but also to better evaluate memorial evidence when making metacognitive judgments. Thus, factors that improve memory performance will also improve metacognitive discrimination between items that will and will not be remembered. In the present context, these frameworks predict that if exercise enhances memory, it will also improve the relative accuracy of metacognitive judgments (i.e., increased discrimination).

Second, exercise could influence the absolute accuracy of metacognitive judgments via a *hard-easy effect*^22,23^. This refers to a well-established phenomenon whereby conditions that impair memory performance are associated with increased overconfidence, and conditions that improve memory performance are associated with reduced overconfidence (or even under confidence). Although the underlying cause of the hard-easy effect has been debated^24,25^, it is a pervasive phenomenon that has been demonstrated in various metacognitive judgments, including prospective JOLs^26^. Thus, if exercise enhances memory performance, there is reason to expect a hard-easy effect; that is, the increase in accuracy caused by exercise will not be accompanied by a commensurate increase in JOLs. In turn, this will likely reduce overconfidence, given that metacognitive judgments tend to be overconfident^27^.

Third, exercise could enhance memory performance without affecting metacognitive accuracy. Several models of metacognition^28–30^ hold that metacognitive judgments are influenced by beliefs about how memory operates and factors that affect memory. Such beliefs might be based on, for example, personal experience, assumptions about memory, or information encountered. Importantly, such beliefs can act as cues for people to adjust their metacognitive judgments in anticipation of memory performance. For example, manipulations that have salient detrimental effects on memory performance can reduce memory performance with negligible effects on the accuracy of metacognitive judgments; when people are aware that their memory is impaired, they can adjust their metacognitive judgments accordingly^31^. Similarly, beliefs hat exercise benefits memory might prompt higher JOLs for items studied under the influence of exercise. As a result, exercise could increase memory performance but leave metacognitive accuracy unaffected.

Finally, there are circumstances under which exercise might impair metacognition by inflating metacognitive judgments relative to memory performance. Although the effects of exercise on memory are generally positive, exceptions occur whereby exercise does not benefit memory performance^3,32^. If exercise does not benefit memory, it may cause an increase in the overconfidence via the mechanisms described in the preceding paragraph. That is, when making JOLs, people may take into account beliefs about the benefits of exercise for memory. This would prompt an increase in JOLs for items studied under the influence of exercise (e.g., before or after exercise). In the absence of an increase in memory performance with exercise, this mechanism would lead people to overestimate memory for items studied under the influence of exercise.

Although no prior research has tested the effects of moderate-intensity exercise on metacognition, two previous studies have examined the effects of low-intensity exercise. Dutton and Carroll^33^ had participants walk on the spot in time to a slow-beating or fast-beating metronome while watching a video. A control group sat instead of walking. Participants later made JOLs about the likelihood of remembering items and events from the video and completed a memory test for that information. Exercise did not influence the accuracy of memory responses or the accuracy of JOLs. Salas, Minakata, and Kelemen^34^ compared conditions in which participants walked briskly for 10 minutes either before or after studying word lists to a condition involving a sitting control task. Participants then made JOLs and completed a memory test for the words. Walking after studying did not influence recall memory or the accuracy of JOLs but walking before studying improved recall performance and reduced the difference between mean JOLs and mean recall performance. These results are consistent with a hard-easy effect.

However, it cannot be assumed that these effects on metacognition will generalize to tasks involving exercise of greater intensity. Effects of exercise on memory are likely to be stronger for moderate intensity exercise than low intensity exercise. For example, in two prominent meta-analyses, the average standardized effect size associated with the effect of exercise on long-term memory is approximately twice as large for moderate intensity exercise as for low intensity exercise^3,4^. Importantly, different effects of exercise on memory might lead to different effects on metacognition. Of the different mechanisms that could underpin effects of exercise on metacognition, several are based on how effects on memory might translate into effects on metacognition (e.g., mechanisms involving the *hard-easy effect* and *optimality hypothesis*). Hence, because the effect of exercise on memory is likely to differ between low and moderate-intensity exercise, and because the effects of exercise on metacognition may well depend on how exercise affects memory, we cannot assume that the effects of exercise on metacognition obtained by Salas *et al*.^34^ and Dutton and Carroll^33^ will also occur for moderate intensity exercise.

To examine the effects of exercise on the accuracy of metacognitive judgments, we conducted two experiments using a paired-associate learning task. Participants studied pairs of words (e.g., *door*-*bowl*), judged the likelihood of recalling the second word of each pair when presented with the first (*door*-?) and completed a cued-recall test in which they attempted to recall the second word of each pair when shown the first. Paired-associate tasks have been used extensively in experiments involving metacognitive judgments^35,36^ and have applied value because they are relevant for many real memory tasks. For example, when learning a new language, words in the native language are paired with the corresponding words from the new language. Similarly, learning definitions involves studying key terms paired with their explanation.

A second aim was to examine the effects of exercise on memory for paired-associates. Exercise generally improves memory.^3,4^ However, in the only such study to investigate memory for paired associates, McNerney and Radvansky^37^ found that exercise before or after studying improved memory for sentences and sequences of spatial locations, but not for word pairs. Our research will add to the data addressing this issue. It is important to note that whether exercise affects memory performance is not crucial for our main objective of studying effects on metacognition, because—as outlined above—exercise might influence metacognition even in the absence of effects on memory.

## Experiment 1

In Experiment 1, participants, we randomly allocated participants to one of three exercise conditions: Exercise before study (*exercise-prior*), exercise after study but before test (*exercise-post*), and a no-exercise control condition (*control*). Participants in the *exercise-prior* group completed a 30-minute exercise task on a stationary bicycle prior to studying word pairs (see Methods for details). Participants in the *exercise-post* group completed the exercise task after studying word pairs and prior to the memory test. To ensure that the retention interval between encoding and test was equivalent between conditions, participants in the exercise-prior and control groups completed a distractor task following the encoding phase (watching a 30-minute video).

Participants studied a list of word pairs (e.g., *door*-*bowl*) and made JOLs for each word pair, rating the likelihood (0–100%) that they would recall the second word when shown the first (e.g., *door-?*). Participants then completed a cued-recall test in which they attempted to recall the second word of each pair when shown the first (*door*-?). We solicited two sets of JOLs, one immediately after the completion of the study phase (JOLS-1) and one immediately before the cued-recall test (JOLs-2). This provided one set of JOLs made soon after the exercise-prior task (JOLs-1) and another made soon after the exercise-post task (JOLs-2), to maximize the detection of any short-lived effects of exercise on metacognition.

Absolute accuracy of JOLs was measured by comparing mean accuracy to mean JOLs^38^. Overconfidence occurs when mean JOLs exceed mean accuracy; under-confidence occurs when mean JOLs fall short of accuracy. Relative accuracy—reflecting participants’ ability to distinguish items that they would correctly remember later from items they would not—was indexed using the Adjusted Normalized Discrimination Index (*ANDI*)^18^. Values of *ANDI* range between zero (*no discrimination*) and one (*perfect discrimination*). Conceptually, the *ANDI* statistic represents the proportion of recall performance accurately predicted by JOLs. We used *ANDI* due to its advantages over other commonly used measures of discrimination. The Goodman-Kruskal gamma correlation is perhaps the most commonly reported statistic for indexing discrimination. However, there are substantial problems with Gamma as a measure of discrimination: It varies systematically with response bias and, hence, Gamma can produce results based on artefacts rather than genuine effects and is more susceptible to Type 1 error than alternative measures^39^.

### Results

#### Heart rate

The heart rate data indicate that the exercise manipulation was successful. Mean heart rate during exercise (pre- and post-) ranged from 128 to 138 beats per minute. Table 1 (upper panel) shows mean heart rate measured at rest, conclusion of the encoding phase, and conclusion of the test phase. The encoding and test phase measures were taken some time after the corresponding exercise task had been completed and, thus, indicate whether the exercise task influenced heart rate for an extended time. We conducted ANCOVA to compare each exercise condition to the control condition after the study phase and after the test phase, controlling for resting heart rate.

**Table 1:** Mean heart-rate in bpm for each exercise condition in Experiments 1 and 2. Standard deviations in parentheses.

	Exercise condition		
	Exercise-prior	Exercise-post	Control
**Experiment 1**			
Rest	79.6 (11.6)	79.2 (14.6)	81.2 (9.3)
After encoding	90.4 (16.3)*	80.2 (15.8)	82.6 (9.1)
After test	78.0 (8.3)	88.2 (15.4)*	78.4 (8.5)
**Experiment 2**			
Rest	78.1 (8.1)	—	72.5 (11.1)
After exercise	112.8 (14.2)*	—	73.2 (12.4)

Note: *Indicates a mean that differed from the control group at the *p* < 0.05 level.

Mean heart rate measured after the study phase (when only the exercise-prior condition had exercised) was greater in the exercise-prior condition than the control condition, *F*(1, 34) = 6.55, *P* = 0.015, Cohen’s *d* = 0.85. There was little difference between the exercise-post and control conditions, *F*(1, 36) < 1, *P* = 0.774, *d* = 0.09. Effect size estimates for these ANCOVA are based on estimated marginal means and associated standard deviations.

Mean heart rate measured after the test phase (when only the exercise-post condition had recently exercised) was greater in the exercise-post condition than the control condition, *F*(1, 36) = 7.99, *P* = 0.008, *d* = 0.91. There was little difference in heart rate between the exercise-prior and control conditions, *F*(1, 36) < 0.1, *P* = 0.958, *d* = 0.02. Thus, the exercise manipulation influenced heart rate as intended.

#### Effects of exercise on cued-recall

Planned comparisons between the control condition and each of the exercise conditions showed that exercise prior to encoding improved memory performance, but exercise after encoding did not. The exercise-prior condition (48.5%) correctly recalled more target items than the control condition (40.0%), *t*(35) = 2.21, *P* = 0.034, *d* = 0.73. There was little difference in recall accuracy between the exercise-post (40.1%) and control conditions, *t*(37) = 0.03, *P* = 0.981, *d* = 0.01.

#### Effects of exercise on the accuracy of JOLs-1

**Figure 1:**
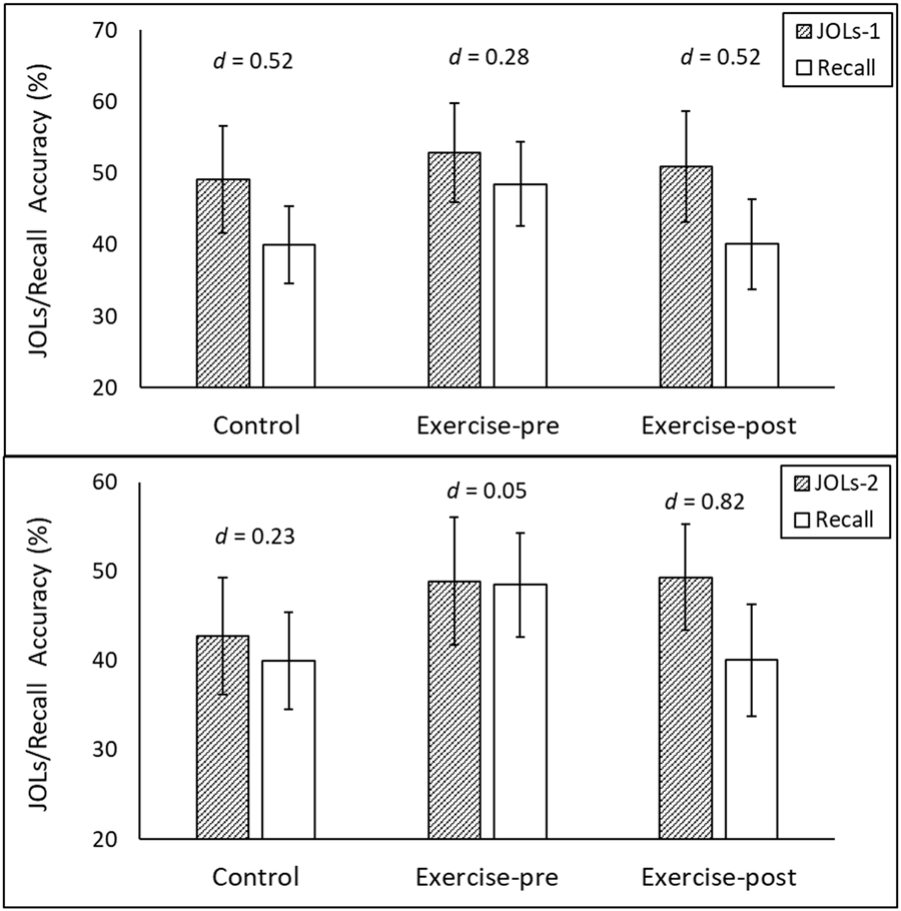
Comparisons between mean cued-recall accuracy and JOLs made immediately after the completion of the study phase (JOLs-1; upper panel) and JOLs made immediately before the cued-recall test (JOLs-2; lower panel) for each exercise condition in Experiment 1. For JOLs-1, the magnitude of overconfidence did not differ significantly between conditions. For JOLs-2, overconfidence was greatest in the exercise-post condition. Cohen’s *d* values indicate standardized effect size estimates. Error bars show 95% confidence intervals.

To assess the absolute accuracy of JOLs made immediately after the completion of the study phase, we conducted a 3 (Exercise condition) × 2 (Measure: JOLs-1, recall accuracy) mixed ANOVA with measure as a within-subjects variable. The dependent measure was percentage recall (either predicted or actual). This analysis provided a comparison between subjective predictions about recall accuracy (JOLs) with actual recall accuracy, an established index of the absolute accuracy of metacognition^29^. JOLs made immediately after the study phase exhibited overconfidence: On average, JOLs-1 (*M* = 51.06, *SD* = 15.53) exceeded recall performance (*M* = 42.92, *SD* = 13.07), *F*(1, 56) = 11.34, *P* = 0.001, *d* = 0.43, (see Fig. 1, upper panel). This pattern did not vary across the different exercise conditions, *F*(2, 56) = 0.67, *P* = 0.508.

To assess the relative accuracy of JOLs-1, we conducted a one-way ANOVA with exercise condition as the independent variable and ANDI values as the outcome. ANDI values did not vary significantly between the control condition and either of the exercise conditions, *F* = 0.59, *P* = 0.355. Additional analyses ruled out floor effects as an explanation: Participants in all of the three exercise conditions demonstrated an ability to distinguish items they could remember from items they could not, evidenced by *ANDI* values greater than zero according to one-sample *t*-tests (all *t* values > 6.1, all *p* values < 0.001). In sum, the exercise manipulation had minimal effect on the absolute or relative accuracy of JOLs-1.

#### Effects of exercise on the accuracy of JOLs-2

To examine the effect of exercise on the absolute accuracy of JOLs made immediately before the cued-recall test, we conducted a 3 (exercise condition) × 2 (measure: JOLs-2, recall accuracy) mixed ANOVA with measure as a within-subjects variable. This yielded an exercise condition × measure interaction, *F*(2, 56) = 3.78, *P* = 0.029, indicating that the discrepancy between JOLs-2 and recall accuracy varied between the exercise conditions (see Fig. 1, lower panel). In the exercise-post condition, JOLs-2 were (on average) higher than recall accuracy, *t*(21) = 3.83, *P* = 0.001, *d* = 0.82. In contrast, JOLs-2 corresponded much more closely to recall in the exercise-pre and control conditions, *t*s < 1, *P*s > 0.36, *d*s < 0.24. Thus, exercising after study produced greater overconfidence for JOLs made immediately before the recall test.

To test the relative accuracy of JOLs-2, we conducted a one-way ANOVA with exercise condition as the independent variable and ANDI values as the outcome. There was minimal difference in *ANDI* values between the experimental conditions, *F* < 0.1, *P* = 0.907. Thus, exercise did not affect participants’ ability to discriminate between items they did and did not recall based on JOLs made just prior to the cued-recall test. This was not due to floor effects: Participants in all three exercise conditions could distinguish items they knew from items they did not, evidenced by non-zero *ANDI* values (all *t* values > 10.4, *P* values < 0.001).

Discrimination was also better for JOLs made immediately before the cued-recall test (JOLs-2) than those made immediately after the study phase (JOLs-1), evidenced by higher *ANDI* values overall (JOLs-2: *M* = 0.47 *SD* = 0.18 vs. JOLs-1: *M* = 0.29, *SD* = 0.17), *F*(1, 56) = 43.67, *MSE* = 0.02, *p* < 0.001, *d* = 0.88. This difference did not vary between exercise conditions (*F* < 0.1). The greater relative accuracy of JOLs-2 may have been due to the longer delay between encoding and JOLs, given that delaying JOLs increases their accuracy^15,35,40^.

Experiment 1 showed that exercise can influence the accuracy of metacognitive judgments. Exercise before encoding did not influence metacognitive accuracy, but exercise after encoding increased overconfidence for JOLs made prior to a cued-recall test. Exercise also affected cued-recall memory performance: Relative to the no-exercise control condition, exercise prior to learning improved cued-recall but exercise after encoding did not. We note that, although post-encoding exercise has sometimes been shown to benefit memory tasks^37^, our results are consistent with other studies in which exercise after study enhanced memory only if there is a delay between study and exercise or between exercise and test^4,7,8^.

## Experiment 2

Experiment 2 extended Experiment 1 in two ways. First, we included a different type of memory task (recognition) and a different metacognitive judgment: *feeling-of-knowing* (FOK). FOK judgments are predictions about the likelihood of recognizing a studied item that cannot currently be retrieved from memory^13^. A high FOK judgment would be made if a person cannot remember the answer to a question now but feels certain of recognizing the answer when seen in the future (e.g., from a list of options). Factors related to memory strength can have different effects on the accuracy of FOK judgments versus JOLs^21,41^; hence, exercise might have different effects on these two types of judgments.

Second, we included an additional control task for the no-exercise condition. Experiment 1 included a control task before the cued-recall test for the conditions that did not involve exercise after encoding, but not a dedicated control task prior to encoding. In Experiment 2, to better equate the experience of the exercise and control groups, participants were randomly allocated to complete either the exercise task or control task (watching a video) before studying word pairs. Because our intention was to test the effects of exercise before study with a more stringent control condition, we omitted the exercise-post condition; participants were randomly allocated to an exercise before study or control condition. For simplicity, we also omitted the measure of JOLs immediately after study (JOLs-1 in Experiment 1) and included only JOLs made immediately before the cued-recall test.

In Experiment 2, participants attended the laboratory and exercised or completed a filler task (watching an animal documentary video) for 30-minutes. Participants then studied a list of word-pairs, made JOLs, and completed a cued-recall test. We solicited one set of JOLs, immediately prior to the cued-recall test. Following the cued-recall test, participants made FOK judgments. Participants were presented with a cue word from the study list (e.g., *cloud*-?) and asked to rate the likelihood of correctly recognizing the corresponding target word from a list of four alternatives (e.g., *spoon; ivory*; *drill*; *choir*). Participants then completed a 4-alternative forced-choice recognition task for the word-pairs. Note that measures related to the recognition test and FOK judgments were based on data from trials for which a correct answer was not provided on the cued-recall test—rather than all trials—because FOK is thought to occur in the absence of memory retrieval^8^ (see Methods for details).

### Results

#### Heart rate

The heart rate data confirmed that the exercise manipulation worked. We conducted an ANCOVA to compare heart rate measured after the exercise manipulation, controlling for resting heart rate measured prior to the manipulation (see Table 1, lower panel). Mean heart rate after the manipulation was higher for the exercise condition than the control condition, *F*(1, 36) = 117.16, *P* < 0.001, *d* = 3.54.

#### Effects of exercise on memory

Exercise before studying did not enhance memory performance; in fact, mean accuracy was numerically higher for the control condition than the exercise condition for both memory tasks. Exercise had no significant effect on the proportion of correct responses for the cued-recall task (Exercise: *M* = 0.12, *SD* = 0.15 vs. Control: *M* = 0.18, *SD* = 0.18), *t*(37) = 1.12, *P* = 0.243, *d* = 0.38, or the recognition memory task (Exercise: *M* = 0.52, *SD* = 0.16 vs. Control: *M* = 0.55, *SD* = 0.15), *t*(37) < 1, *P* = 0.513, *d* = 0.21.

#### Effects of exercise on the accuracy of JOLs

**Figure 2:**
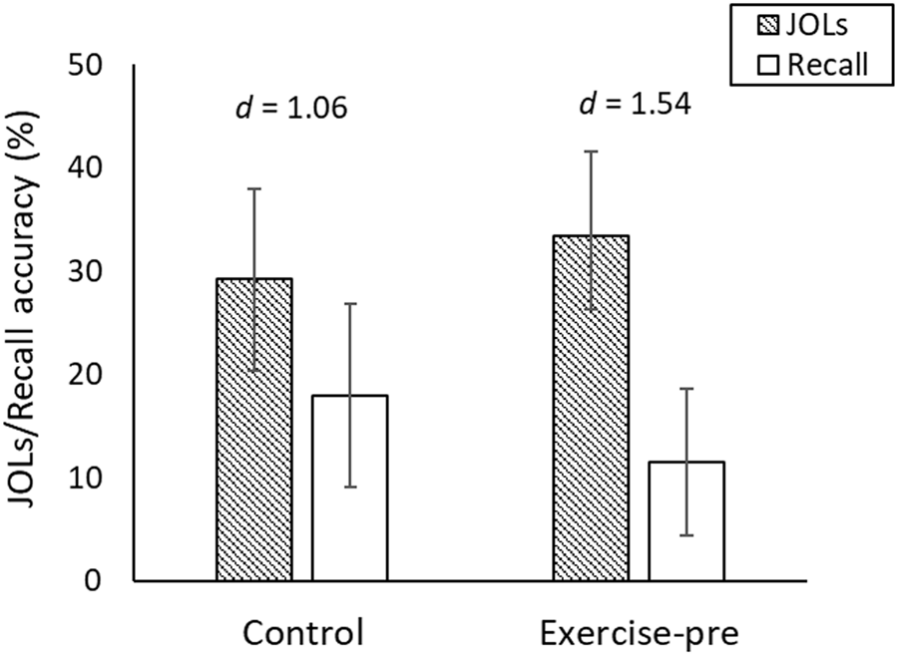
Mean cued-recall accuracy and JOLs for each exercise condition in Experiment 2. Effect size estimates (Cohen’s d) reflect the degree of overconfidence in each condition. Error bars show 95% CIs.

To examine the absolute accuracy of JOLs, we conducted a 2 (exercise condition) × 2 (measure: JOLs, recall accuracy) mixed ANOVA, with measure as a within-subjects factor^29^. Exercise affected the accuracy of JOLs, evidenced by an interaction between exercise condition and measure, *F*(1, 37) = 6.74, *P* = 0.013. As shown in Fig. 2, JOLs were overconfident in both conditions, but the discrepancy between JOLs and cued-recall accuracy was greater in the exercise condition than the control condition. Simple effects analyses supported this conclusion: JOLs exceeded accuracy in the exercise condition, *t*(19) = 6.82, *P* < 0.001, and control conditions, *t*(19) = 4.61, *P* < 0.001, but the associated effect sizes indicated that overconfidence was greater in the exercise (*d* = 1.54) than the control condition (*d* = 1.06).

Exercise also impaired the relative accuracy of JOLs, with lower *ANDI* values for the exercise group (*M* = 0.43, *SD* = 0.30) than the control group (*M* = 0.69, *SD* = 0.18), *t*(30.01) = 3.09, *P* = 0.004, *d* = 1.01. Thus, exercise reduced the extent to which participants could discriminate items they would recall from items they would not recall. This effect was unanticipated and prompted exploratory analyses to investigate why it occurred.

#### Exploratory analyses

One possibility—not considered *a priori*—is that the effects exercise may vary depending on memory strength for individual items. Various theoretical accounts of metacognition suggest that memory strength influences the extent to which metacognitive judgments are based on mnemonic cues (e.g., the ability to retrieve information about the target item)^21,22,42,43^. When memory is weaker, mnemonic cues are less likely to be available, and metacognitive judgments might be based on other types of cues (e.g., beliefs about the benefits of exercise for memory).

To test this possibility, we compared the effects of exercise on JOLs for items that participants later remembered correctly on the cued-recall test versus items that participants did not remember correctly, via a 2 (exercise condition) × 2 (accuracy of cued-recall response) mixed ANOVA with accuracy as a within-subjects variable and mean JOLs as the outcome variable. The effect of exercise on JOLs varied depending on accuracy on the cued-recall test, *F*(1,31) = 4.79, *P* = 0.036. For items that were not remembered on the cued-recall test, JOLs were higher in the exercise condition (*M* = 26.38, *SD* = 14.39) than the control condition (*M* = 16.46, *SD* = 12.72), *t*(37) = 2.28, *P* = 0.029, *d* = 0.73. For items that were remembered correctly on the cued-recall test, there was little difference in JOLs between the exercise (*M* = 86.95, *SD* = 14.58) and control conditions (*M* = 89.82, *SD* = 8.70), *t* < 1, *P* = 0.518, *d* = 0.23. These results are consistent with the idea that the influence of exercise-related cues on JOLs was greater when memory was weaker. Importantly, this explanation can account for the effect of exercise on discrimination: Exercise selectively increased JOLs for non-remembered items, reducing the difference in JOL magnitude between remembered and non-remembered items on the test, which—in turn—reduced discrimination. We emphasize that this explanation, although plausible, was not considered *a priori*.

#### Effects of exercise on the accuracy of FOK judgments

**Figure 3:**
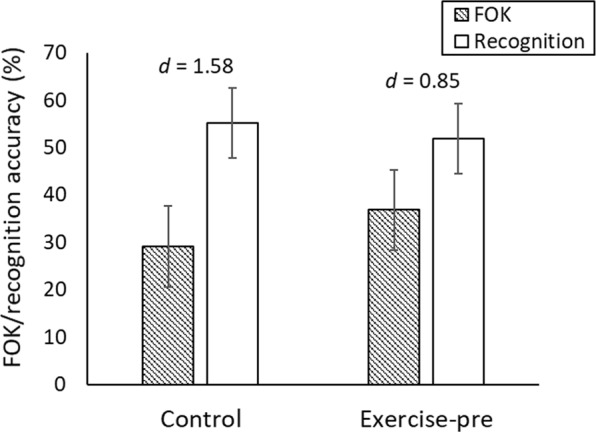
Mean recognition accuracy and FOK judgments for each exercise condition in Experiment 2. Effect size estimates (Cohen’s *d*) reflect the degree of under-confidence in each condition. Error bars show 95% CIs.

The effects of exercise on FOK judgements were less clear-cut. In terms of absolute accuracy, FOK judgments were lower than recognition accuracy rates in both exercise conditions, indicating under-confidence (see Fig. 3). The discrepancy between FOK and accuracy was, if anything, larger in the control condition, *t*(18) = 6.86, *P* < 0.001, *d* = 1.58, than the exercise condition, *t*(19) = 3.77, *P* = 0.001, *d* = 0.85, suggesting that exercise reduced under-confidence in FOK judgments. However, the interaction between exercise condition and measure (FOK, accuracy) was not statistically significant, *F*(1, 37) = 3.95, *P* = 0.054.

Exercise had minimal effect on the relative accuracy of FOK judgments, with little difference in ANDI between the exercise (*M* = 0.04, *SD* = 0.05) and control groups (*M* = 0.05, *SD* = 0.07), *t*(37) < 1, *P* = 0.600, *d* = 0.17.

Experiment 2 produced no evidence that exercise before encoding benefited memory performance. Exercise did, however, affect the absolute accuracy of metacognitive judgments. Exercise before encoding increased perceived likelihood of remembering, relative to actual likelihood of remembering, but the consequences of this effect differed between JOLs and FOK judgments. Compared to the control condition, exercise reduced the absolute accuracy of JOLs by increasing overconfidence, whereas exercise increased the absolute accuracy of FOK judgments by reducing under-confidence. Exercise also affected the relative accuracy of JOLs: Compared to control participants, those who exercised were less able to use JOLs to distinguish between items they would and would not remember on the cued-recall test.

## General Discussion

**Figure 4:**
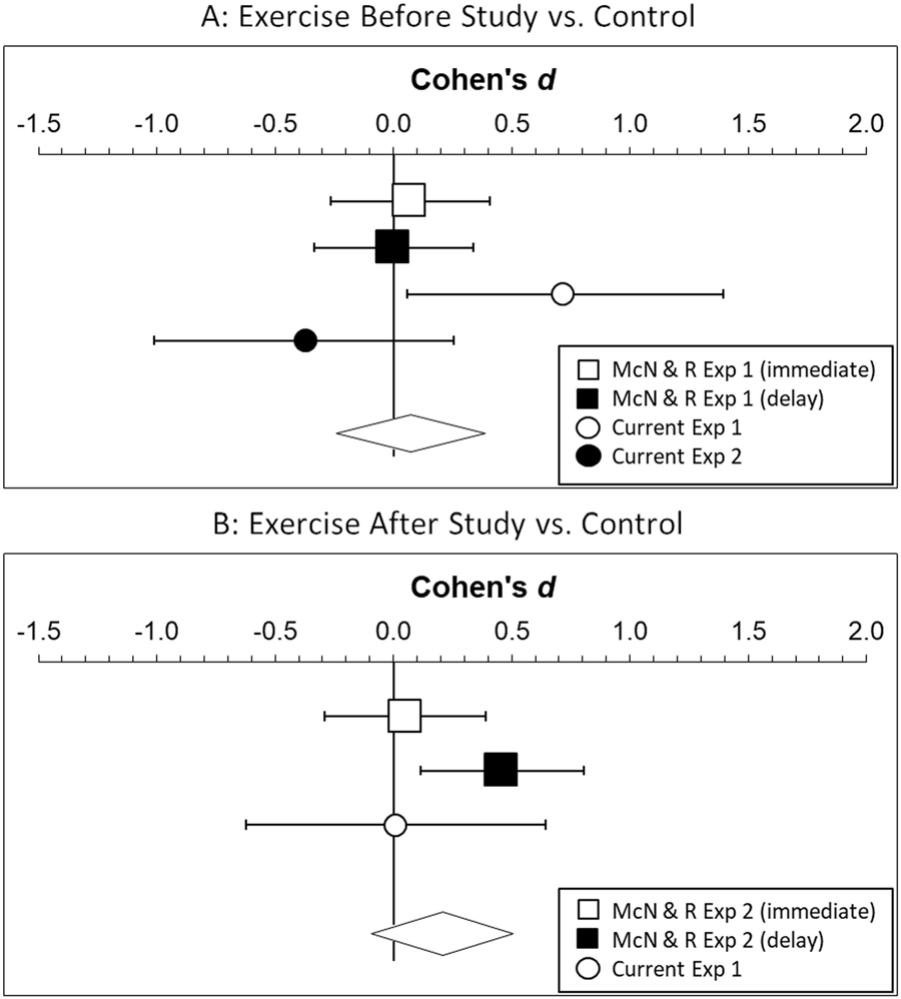
Forest plots of Cohen’s *d* effect sizes for comparisons of cued-recall memory performance in exercise vs. control conditions in McNerney & Radvansky^37^ and the current experiments. Error bars show 95% confidence intervals (95% CIs). The diamond at the bottom of each panel indicates the meta-analytic effect size and 95% CIs.

The results of these experiments shape our understanding of the effects of exercise on cognition and metacognition in several ways. First, the results advance knowledge about the effects of exercise on memory for paired associates. The few experiments to test the effects of acute exercise on memory for paired associates (the current experiments and McNerney and Radvansky’s^37^) have produced discrepant results. To further investigated this issue, we conducted a meta-analysis of effect sizes across the individual experiments in our research and McNerney and Radvansky’s^37^ using Exploratory Software for Confidence Intervals (ESCI)^44,45^. The results appear in Fig. 4. For exercise before encoding, the meta-analytic effect size across all experiments was trivial and non-significantly different from zero, *d* = 0.07, 95% CI [−0.24, 0.39], *t* = 0.46, *p* = 0.645. Thus, across all relevant data, exercise before study does not seem to enhance memory for paired associates.

For exercise after encoding, the meta-analytic effect size was small and non-significantly different from zero, *d* = 0.21 [−0.09, 0.51], *t* = 1.36, *p* = 0.175. This suggests that exercise after encoding is unlikely to benefit memory for paired associates. However, it is important to note that the results in Fig. 4 (panel B) are consistent with the notion that effects of post-encoding exercise are stronger when there is a delay between either study and exercise or exercise and test, as shown for other memory tasks^4,7^. Thus, without further data, it would not be appropriate to draw confident conclusions about the effects of exercise after encoding on memory for paired associates.

In our study, exercise before encoding enhanced memory performance in Experiment 1 but not Experiment 2. This difference was not anticipated but might have arisen due to the difference in overall memory performance between the two experiments. McNerney and Radvansky^37^ suggested that beneficial effects of exercise on paired associate learning might emerge under conditions that involve deeper processing, producing stronger memory (e.g., repeated study-test cycles; generation of responses during study phases^12^). Our data offer some support for this notion: Exercise benefitted cued-recall in Experiment 1 but not Experiment 2, and overall cued-recall performance was better in Experiment 1 (.43 correct) than Experiment 2 (.15 correct). Thus, the effects of exercise before study on memory for paired associates might be moderated by strength of memory; future research may shed further light on this issue. As an aside, we suggest that the superior cued-recall performance in Experiment 1 might be due to the inclusion of a second JOL measure. Making a delayed JOL invites a covert attempt to retrieve the target word when presented with the cue (*door-?*)^15^. Hence, an extra JOL measure provided an extra opportunity for retrieval practice which, in turn, consistently benefits memory^46^.

The second main contribution of these results is the demonstration that a bout of acute exercise can affect the absolute accuracy of metacognitive judgments. It is important to interpret this finding in light of similarities and differences in results for the two experiments and the different types of metacognitive judgments. In terms of similarities, across both studies and both measures of metacognition, our data indicate that when exercise does not benefit memory (relative to a no-exercise control group) it can increase predictions about memory performance relative to actual performance. This occurred for exercise post-encoding in Experiment 1 and exercise pre-encoding in Experiment 2 and applied to JOLs and FOK judgments. In contrast, when exercise benefitted cued-recall performance (exercise pre-encoding in Experiment 1), there was no effect on the absolute accuracy of metacognition. Thus, the two experiments provide converging evidence that exercise can increase predictions of memory performance relative to actual memory performance, under conditions whereby exercise does not benefit memory.

In terms of differences in results, when exercise did not benefit memory, the effects on the absolute accuracy of metacognition varied depending on the type of metacognitive judgment and—importantly—whether judgments tended to be over- or under-confident in the absence of exercise. For JOLs, judgments tended to be overconfident, and this overconfidence was increased by exercise post-encoding (Experiment 1) and pre-encoding (Experiment 2). In contrast, FOK judgments (Experiment 2) tended to be under-confident, and this under-confidence was reduced by pre-encoding exercise, resulting in greater absolute accuracy. This difference in the effects of exercise on the absolute accuracy of metacognitive judgments likely stemmed from differences in memory accuracy between the cued-recall and recognition tests. As noted in the Introduction, metacognitive judgments tend to be more overconfident under conditions where memory accuracy is worse (the hard-easy effect)^22^. In our research, participants tended to overestimate future remembering on cued-recall tests (which produced relatively low accuracy) and underestimate remembering on the recognition test (higher accuracy). Thus, when exercise increased predicted remembering, this manifested as an increase in overconfidence for predictions about cued-recall and a reduction in under-confidence for predictions about recognition.

However, the entire pattern of results cannot be explained by the hard-easy effect, whereby differences in the absolute accuracy of metacognitive judgments accompany differences in memory performance. Here, differences in metacognitive accuracy occurred in the absence of differences in memory performance. The effects are, however, consistent with the notion that exercise increased subjective likelihood of remembering items on a future test, relative to actual likelihood of remembering. Two types of mechanisms could underpin this effect. First, exercise might prompt adjustments to metacognitive judgments based on belief-based cues^30^ about exercise enhancing memory, a possibility is made more plausible by the prominence of recent popular media articles about the cognitive benefits of exercise^47,48^. Second, exercise could provide experience-based cues for future remembering^38,49^. For example, acute exercise often produces subjective states of increased alertness, refreshment, and calmness^50–52^ which could increase perceived likelihood of remembering. Our data do not allow us to adjudicate between belief- and experience-based mechanisms, but recent evidence suggests that both likely contribute to metacognitive judgments^53^.

Third, an acute bout of exercise can affect the relative accuracy of metacognitive judgments. In Experiment 2, pre-encoding exercise impaired participants’ ability to distinguish between items they would and would not remember on a cued-recall test. Exploratory analyses showed that this occurred because exercise selectively increased JOLs for items that participants failed to remember. As explained in the Discussion for Experiment 2, this pattern of results is consistent with theories that hold that metacognitive judgments are more susceptible to non-mnemonic cues when memory is weak^21,22,42,43^. However, these results must be interpreted with caution because they were not considered *a priori*.

Finally, our results have implications for applied settings, such as students needing to accurately gauge their memory for paired associates in learning tasks, including learning definitions and new languages. Our results show that moderate-intensity exercise around the time of study can impair JOLs for paired-associates. To be clear, we do not suggest that people should avoid exercise for the sake of metacognition. There is overwhelming evidence that ongoing exercise has long-term benefits for health and cognition^1,2,54^. Our data in no way challenge these conclusions. However, our results do suggest there is benefit in developing strategies for incorporating acute exercise into study routines in ways that facilitate learning without undermining metacognition. For example, simple interventions such as hypothesis disconfirmation or delaying JOLs for longer after exercise, may prove effective in mitigating any tendency for exercise to inflate JOLs^55,56^.

## Methods

### Ethical approval

This project was approved by the University of Tasmania Human Research Ethics Committee (Reference H0014950) and carried out in accordance with relevant guidelines and regulations. Informed consent was obtained from all participants.

### Experiment 1

#### Participants

Fifty-nine adults (33 female; 28 male) aged 18 to 62 years (*M* = 25.75, *SD* = 8.31) were recruited from the University of Tasmania and the wider community of Northern Tasmania. Note that we did not restrict the age range of participants because the benefits of exercise on memory are promising for a variety of ages^5^. First-year psychology students received course credit and other participants received an honorarium of $AUD30.

We used the Adult Pre-Exercise Screening Tool^57^ to screen symptoms or conditions necessitating exclusion from the study (e.g., cardiovascular disease, physical injuries). Participants were randomly allocated to the *no exercise* (*n* = 17), *exercise-prior* (*n* = 20), or *exercise-post* condition (*n* = 22). The three groups did not differ in mean age or BMI (*F* values < 1.30, *p* values > 0.28). Two additional participants were excluded from analyses due to minimal variation in JOLs. One gave JOLs of 50% for every word pair; the other gave JOLs of 50% for 89 of the 90 word-pairs (JOLs-2).

#### Materials and procedure

The testing session took approximately two hours. All participants completed a memory task involving encoding and test phases. Participants completed a 30-minute exercise task prior to the encoding phase (*exercise-prior* group), between the encoding and test phases (*exercise-post* group), or not at all (*control* group). Participants in the exercise-prior and control groups completed a distractor task following the encoding phase, to ensure that the retention interval between encoding and test was equivalent between conditions. This involved watching a 30-minute documentary video about animals, chosen to minimize any chance of producing an emotional or arousal reaction.

Participants in the post-exercise and control conditions did not complete a dedicated control task prior to encoding; participants in these conditions arrived at the laboratory and received initial instructions before beginning the encoding phase.

#### Exercise task

Participants in the exercise condition completed the exercise task on a cycle ergometer (Ergomedic 828E, Monark Exercise AB, Vansbro Sweden). The task involved a 5-minute warm-up, 20-minutes of moderate-intensity exercise, and a 5-minute cool down. Prior to the task, participants were instructed that during the moderate-intensity exercise phase they should experience the feeling of strain and fatigue in their muscles, and changes to their breathing but not to an extent that undermines their ability to hold a conversation. This corresponds to a level of 3–4 on the Rating of Perceived Exertion (RPE) scale^58^ which ranges from 0 (sedentary) to 11 (maximal physical exertion).

During the moderate-intensity exercise phase, participants were asked to maintain a pedaling cadence of 60 RPM and regulate the resistance of the ergometer to keep their subjective exertion at a level of 3–4 on the RPE scale. Participants reported their RPE at 5-min intervals during the exercise task. Participants were provided with water to prevent dehydration.

Heart rate sensors were worn just below participants’ sternums throughout the study. Heart rate was recorded at the beginning of the study, the end of the encoding and test phases, and every five minutes during the 20-minute exercise task for participants in the two exercise groups. A target range for heart rate was calculated for each participant by subtracting the participant’s age from 220^57,59^. Participants were instructed to adjust the resistance of the ergometer if their heart rate moved outside of their target range. Mean heart rate was between 128 and 138 beats per minute, and the patterns did not differ between the exercise-prior and exercise-post conditions, or across any of the time points during exercise, *F* values < 1.06, *P* values > 0.310.

#### Memory and metacognition tasks

Participants studied 90 English-English word pairs (e.g., *glass*-*petal*) displayed on a 19-inch PC screen in randomized order. Word-pairs were displayed one-at-a-time and progress between pairs was self-paced. The word-pairs were taken from Nelson, McEvoy, and Schreiber^60^. To generate variance in difficulty, half of the pairs were unrelated (e.g., *throw-city*) making them relatively difficult to remember, whereas the others were associated (e.g., *wool-lamb*) making them relatively easy to remember (associative strength scores ranged from 0-0.88)^60^.

Participants provided two sets of JOLs. The first set occurred after completing the study phase (JOLs-1). After the presentation of all word pairs, participants made JOLs for each word pair. Participants were shown the first word of each pair (e.g., *glass*-?) and asked to predict the likelihood that they would be able to recall the second word when shown the first (on a scale from 0–100%). The second set of JOLs occurred immediately before the cued-recall test, and followed the same procedure (JOLs-2). This provided a set of JOLs measured soon after completion of the exercise-prior task and another set measured soon after the completion of the exercise-post task. Note that if we collected only JOLs-1, the exercise-post group would not make JOLs under the influence of exercise; if we collected only JOLs-2, there would be a long delay between study and JOLs for the exercise-prior group.

On each trial of the cued-recall test, participants saw a cue word (e.g., *glass*-?) and were asked to recall the corresponding target. This procedure was repeated for each word-pair, in randomized order. Misspelled items (e.g., *tounge* instead of *tongue*) or variations of a single item (e.g., *card*, *cards)* were scored as correct.

### Experiment 2

#### Participants

Thirty-nine adults (20 females) aged 18 to 40 years (*M* = 22.9 years, *SD* = 5.5) who did not complete Experiment 1 were recruited from the University of Tasmania and the wider community of Northern Tasmania. Participants were randomly allocated to an exercise (*n* = 20) or control condition (*n* = 19). First-year psychology students received course credit and other participants received an honorarium of $AUD30. Health screening protocols were the same as Experiment 1. Two additional participants were excluded from analyses (one provided JOLs of zero for all 100 word-pairs; the other provided JOLs of zero for 95 word-pairs). The exercise and control groups did not differ in mean age or BMI (*F* values < 1.30, *p* values > 0.262).

#### Exercise task

Participants in the exercise condition completed the same exercise task as used in Experiment 1, involving 30-min of moderate-intensity exercise before beginning the study phase of the memory task. Participants in the control condition watched a 30-minute neutral video (an animal documentary) before beginning the study phase.

#### Memory and metacognition tasks

The study phase was the same as Experiment 1, except that a different set of 100 English-English word-pairs was used to increase generalizability of results. After all word-pairs had been studied, participants made JOLs for each word pair. For each JOL, the first word of that pair was presented on screen and participants rated the likelihood they would recall the second word when shown the first word on a future test, on a scale from 0% - *completely uncertain* to 100% - *completely certain*.

All participants then completed a 20-min distractor task (viewing an animal documentary video) before beginning the cued-recall test. As in Experiment 1, on each trial, participants saw the first word of each studied pair and attempted to recall the second word. Following the cued-recall test, participants made FOK judgments. Participants were shown the first word of each word pair (e.g., *glass*-?) and asked to rate the likelihood that they would be able to correctly recognize the corresponding word of that pair from a list of four alternatives (on a scale from 0% - *completely uncertain* to 100% - *completely certain*).

Participants then completed a recognition test. On each trial, participants were shown the first word of each pair (e.g., *glass*-?), and attempted to recognize the second word from a list of four alternatives (e.g., *coral; witch*; *petal*; *tunic*). The correct answer was always present among the alternatives, and we counterbalanced the position of the response alternatives such that, across the whole test, the correct answer was equally likely to appear in each of the four positions.

The procedure for collecting and analysing FOK data was based on that used in previous research^61^. Participants made FOK judgments for all word pairs, not just the ones they failed to recall. This allowed participants to make FOK judgments without receiving feedback about the accuracy of their cued-recall responses. Because FOK is thought to occur in the absence of retrieval^14^, analyses of recognition data, FOK judgments, and indices of metacognition associated with FOK judgments (*ANDI*) were based only trials for items that were not correctly answered on the cued-recall test. Analysis including all trials—rather than only items that were not correctly answered on the cued-recall test—produced very similar recognition results but with accuracy rates approximately 5% higher.

## Data Availability

The datasets generated during and/or analysed during the current study are available from the corresponding author on reasonable request.
